# Nanaomycin K inhibited epithelial mesenchymal transition and tumor growth in bladder cancer cells in vitro and in vivo

**DOI:** 10.1038/s41598-021-88741-3

**Published:** 2021-04-28

**Authors:** Koichi Kitagawa, Katsumi Shigemura, Aya Ishii, Takuji Nakashima, Hirotaka Matsuo, Yoko Takahashi, Satoshi Omura, Jun Nakanishi, Masato Fujisawa

**Affiliations:** 1 Department of Advanced Medical Science, Kobe University Graduate School of Science, Technology and Innovation, 7-5-1 Kusunoki-cho, Chuo-ku, Kobe, 650-0017 Japan; 2 Department of Public Health, Kobe University Graduate School of Health Sciences, 7-10-2 Tomogaoka, Suma-ku, Kobe, 654-0142 Japan; 3 Department of Urology, Kobe University Graduate School of Medicine, 7-5-2 Kusunoki-cho, Chuo-ku, Kobe, Hyogo 650-0017 Japan; 4 Kitasato Institute for Life Sciences, Kitasato University, 5-9-1 Shirokane, Minato-ku, Tokyo, 108-8641 Japan; 5 Graduate School of Infection Control Sciences, Kitasato University, 5-9-1 Shirokane, Minato-ku, Tokyo, 108-8641 Japan; 6 Research Center for Functional Materials, National Institute for Materials Science (NIMS), 1-1 Namiki, Tsukuba, Ibaraki 305-0044 Japan

**Keywords:** Cancer, Urology

## Abstract

Nanaomycin K, derived from *Streptomyces rosa* subsp. *notoensis* OS-3966^T^, has been discovered to have inhibitory bioactivity on epithelial–mesenchymal transition (EMT), an important mechanism of cancer cell invasion and migration. In this study, we examined the anti-EMT and anti-tumor effect of nanaomycin K in bladder cancer, where EMT has important roles in progression. We treated two bladder cancer lines, non-muscle-invasive KK47 and muscle-invasive T24, with nanaomycin K to determine the effects on cell proliferation, apoptosis and expression of EMT markers in vitro. Wound-healing assays were performed to assess cell invasion and migration. We conducted an in vivo xenograft study in which mice were inoculated with bladder cancer cells and treated with intratumoral administration of nanaomycin K to investigate its anti-tumor and EMT inhibition effects. As the results, nanaomycin K (50 µg/mL) significantly inhibited cell proliferation in KK47 (*p* < 0.01) and T24 (*p* < 0.01) in the presence of TGF-β, which is an EMT-inducer. Nanaomycin K (50 µg/mL) also significantly inhibited cell migration in KK47 (*p* < 0.01) and T24 (*p* < 0.01), and induced apoptosis in both cell lines in the presence of TGF-β (*p* < 0.01). Nanaomycin K increased the expression of E-cadherin and inhibited the expression of N-cadherin and vimentin in both cell lines. Nanaomycin K also decreased expression of Snail, Slug, phospho-p38 and phospho-SAPK/JNK especially in T24. Intratumoral administration of nanaomycin K significantly inhibited tumor growth in both KK47 and T24 cells at high dose (1.0 mg/body) (*p* = 0.009 and *p* = 0.003, respectively) with no obvious adverse events. In addition, nanaomycin K reversed EMT and significantly inhibited the expression of Ki-67 especially in T24. In conclusion, we demonstrated that nanaomycin K had significant anti-EMT and anti-tumor effects in bladder cancer cells, suggesting that nanaomycin K may be a therapeutic candidate for bladder cancer treatment.

## Introduction

Nanaomycins were isolated from a cultured broth of actinomycete strain *Streptomyces rosa* subsp. *notoensis* OS-3966^T^, known to produce 11 nanaomycin compounds (Nanaomycin A–E and F–J)^1,2^. In our previous study, we found that nanaomycin H, an 8th analog of nanaomycin, selectively killed mechanically and chemically-induced epithelial–mesenchymal transition (EMT) driven Madin–Darby canine kidney (MDCK) cells in vitro^3^. Nanaomycin K, an 11th analog of nanaomycin, was discovered from the culture broth of strain OS-3966 and showed higher bioactivity and cytotoxicity against MDCK cells and inhibition of EMT induced by transforming growth factor (TGF)-β1 compared to nanaomycin H in vitro^4^.

EMT is an important mechanism whereby cancers cells acquire the abilities of invasion and migration. EMT involves the loss of E-cadherin and increased expression of several transcriptional repressors of E-cadherin (Zeb-1, Zeb-2, Twist, Snail, and Slug) and overexpression of N-cadherin and vimentin, with resulting changes that lead to decreased cell adhesion and loss of polarity and tight cell junctions^5^. At the same time, epithelial cells acquire mesenchymal phenotypes, notable motility and capability for invasion and metastasis^5^. Transforming growth factor (TGF)-β is a secreted cytokine that promotes cell invasion and migration, and also promotes EMT in many cancer types including breast cancer, gastric cancer and bladder cancer, resulting in metastasis and chemotherapy resistance^6^.

In the progression of bladder cancer, tumor invasiveness is an important factor for patient outcomes and for deciding on therapeutic options. Muscle-invasive cancer has limited therapeutic options and once metastasis occurs only chemotherapeutic treatments and immune checkpoint inhibitors are currently available^7^ EMT has important roles in the progression of bladder cancer in from non-muscle invasive to muscle-invasive, resulting in metastasis and poor clinical outcome^8^. Mesenchymal phenotypes are also correlated with poor prognosis and associated with resistance to chemotherapy^9^. Therefore, EMT-targeted therapy is a rational therapeutic possibility for treating invasive bladder cancers.

In this study, we examined the anti-tumor effect of nanaomycin K on inhibition of EMT in bladder cancer, as a potential therapeutic candidate for muscle-invasive bladder cancer in vitro and in vivo. We also compared the bioactivity in non-muscle invasive and muscle-invasive cancer to evaluate EMT inhibition.

## Materials and methods

### Cells and reagents

Two human urothelial carcinoma cell lines, non-invasive KK47 (Cell Resource Center for Biomedical Research Institute of Development, Aging and Cancer, Tohoku University, Miyagi, Japan) and invasive T24 (American Type Culture Collection, Manassas, VA), were cultured in RPMI-1640 medium supplemented with 10% fetal bovine serum (Sigma-Aldrich, St. Louis, MI), 1% penicillin and streptomycin at 37 °C and 5% CO_2_^10^. Nanaomycin K was isolated from a cultured broth of *S. rosa* subsp. *notoensis* OS-3966^T^ and purified as previously described^4^. Nanaomycin K was dissolved and diluted with dimethyl sulfoxide (DMSO).

### Cell proliferation assay

We performed cell proliferation assays using KK47 and T24 in the presence of nanaomycin K to determine anti-tumor bioactivity in vitro. Two thousand KK47 and T24 cells were seeded for 24 h and then divided into 3 groups and switched to media^10^ with or without 5 ng/mL TGF-β (FUJIFILM Wako Pure Chemicals, Osaka, Japan), or 5 ng/mL TGF-β^11^ and 36 ng/mL SB-431542 (FUJIFILM Wako Chemicals), a TGF-β receptor inhibitor. After switching media, two different concentrations of nanaomycin K (5 µg/mL, 50 µg/mL) or DMSO (0.05%, 0.5%) were added to the cultures. After incubation for 0, 24, 48, 72 and 96 h, cell proliferation was measured by using 3-(4, 5-dimethylthiazol-2-yl)-5-(3-carboxymethoxyphenyl)-2-(4-sulfophenyl)-2H-tetrazolium (MTS) (Promega Corporation, Madison, WI) according to the manufacturer’s instructions. All experiments were carried out in triplicate.

### Wound healing assay

We performed cell proliferation assays using KK47 and T24 in the presence of nanaomycin K to determine anti-tumor bioactivity in vitro. 1 × 10^5^ cells were seeded and incubated overnight and then divided into 2 groups and switched to media with or without 5 ng/mL TGF-β for 24 h. After incubation, nanaomycin K (5 µg/mL or 50 µg/mL) or 0.05% DMSO were added to the culture. After incubation for 48 h, cells were incubated with 10 µg/mL mitomycin C for 2 h to suppress cell proliferation. After washing the cells with medium, cell monolayers were scratched by 200 µL pipette tips and then washed and incubated with fresh medium for an additional 40 h. Microscopic images were taken at time points 0, 18, 24 and 40 h after scratching. Wound closure was calculated by following formula: (wound area at 0 h − wound area at time)/(wound area at 0 h) × 100. All experiments were carried out in quadruplicate.

### Real time RT-PCR for EMT markers

We performed real time RT-PCR to determine the gene expression of EMT markers E-cadherin, N-cadherin and vimentin in the presence of nanaomycin K in the tested bladder cancer cell lines. 1 × 10^5^ KK47 and T24 cells were seeded and incubated overnight, and then divided into 2 groups and switched to media with or without 5 ng/mL TGF-β. After incubation for 24 h, 5 µg/mL nanaomycin K or 0.05% DMSO was added to the cultures. After incubation for an additional 48 h, cells were collected and total RNA was extracted using NucleoSpin RNA (TaKaRa Bio, Inc, Kusatsu, Japan). Then cDNAs were synthesized by reverse transcription of the extracted total RNAs with a PrimeScript RT reagent kit with gDNA Eraser (TaKaRa Bio, Inc), and real-time RT-PCR assays were performed with primer sets as described in Table 1, TB Green Premix Ex TaqII (TaKaRa Bio, Inc) and Thermal Cycler Dice Real Time System (TaKaRa Bio, Inc). Data analysis was performed by the ^ΔΔ^Ct method^12^.

**Table 1:** Primers used for real time RT-PCR.

Genes		Sequences (5′–3′)
*E-cadherin*	Forward	CAAATCCAACAAAGACAAAGAAGGC
	Reverse	ACACAGCGTGAGAGAAGAGAGT
*N-cadherin*	Forward	CATCATCATCCTGCTTATCCTTGT
	Reverse	GGTCTTCTTCTCCTCCACCTTCT
*Vimentin*	Forward	TCGTGATGCTGAGAAGTTTCG
	Reverse	TCTGGATTCACTCCCTCTGGT
*β-actin*	Forward	CTTAGTTGCGTTACACCCTTTCTTG
	Reverse	CTGTCACCTTCACCGTTCCAGTTT

### Western blotting

Cells were seeded and incubated overnight, and then divided into 2 groups and switched to media with or without 5 ng/mL TGF-β. After incubation for 24 h, nanaomycin K (5 µg/mL or 50 µg/mL) or 0.05% DMSO was added to the cultures. After incubation for an additional 48 h, cells were washed and lysed in 8 M urea buffer. Each sample was added into sample buffer (Nacalai Tesque, Kyoto, Japan) and heated at 95 °C for 5 min. The samples were separated by SDS-PAGE and transferred to PVDF membranes. After blocking with Blocking One or Blocking One-P (Nacalai Tesque) followed by washing, the membranes were incubated overnight at room temperature with anti-E-cadherin (Biolegend, San Diego, CA), anti-N-cadherin (Biolegend), anti-vimentin (Biolegend), anti-phospho-p38 MAPK (Thr180/Tyr182) (Cell Signaling Technology: CST, Danvers, MA), anti-phospho-SAPK/JNK (Thr183/Tyr185) (CST), anti-phospho-p44/42 MAPK (Erk1/2) (Thr202/Tyr204) (CST), anti-Snail (CST), anti-Slug (CST) or anti-β-actin (Santa Cruz Biotechnology, Dallas, TX), respectively. After another washing, membranes were incubated for 1 h with HRP-conjugated secondary antibodies. Antibody binding to proteins was detected by enhanced chemiluminescence.

### Detection of apoptosis

To investigate the mechanism of inhibitory effect of nanaomycin K on cell proliferation, 1 × 10^5^ cells were seeded for 24 h, then switched to media containing 5 ng/mL TGF-β. After incubation for 24 h, nanaomycin K (5 µg/mL or 50 µg/mL) or DMSO (0.05% or 0.5%) was added to the cultures. After incubation for 48 h, cells were stained by Annexin V-FITC and PI (Nacalai Tesque) according to the manufacturer’s instructions. Apoptotic cells were determined by flow cytometry using the Guava easyCyte cytometer (Luminex, Austin, TX) and analyzed by InCyte software (version 3.1) (Luminex).

### Animal experiments

Animal experiments using a mouse bladder cancer model were done to investigate the anti-tumor effects of nanaomycin K. Male 6–8 week-old BALB/c nu/nu mice were purchased from CLEA Japan, Inc (Tokyo, Japan). 1 × 10^6^ cells were inoculated at day 0 (n = 4, respectively) with Matrigel (Corning, Corning, NY). Mice were randomly assigned to 2 treatment groups (0.5 mg/body and 1.0 mg/body of nanaomycin K) and control groups (DMSO). Each dose of nanaomycin K was intratumorally administered with Gelform (Pfizer, New York, NY). Tumor volume was expressed by the following formula: (longest diameter) × (shortest diameter)^2^ × 0.5. Nine days after treatment, mice were sacrificed and tumors were collected^10^.

### Immunohistochemical staining

Tumors were fixed and embedded with paraffin. Paraffin-embedded tissue sections were deparaffinized and rehydrated. Antigen retrieval was performed in citrate buffer (pH 6.0) at 120 °C for 5 min. Immunohistochemical staining (IHC) was performed in an automatic tissue processor (Bond-Max; Leica Microsystems, Wetzlar, Germany) following the standard protocol. Briefly, tissue sections were incubated with the following primary antibodies: anti-E-cadherin, anti-N-cadherin, anti-vimentin, and anti-Ki-67^10^. After washing, sections were exposed to HRP-conjugated secondary antibodies, according to the instrument’s standard protocols. Tissue sections were incubated with diaminobenzidine and counterstained with hematoxylin. The resulting tissue slides were observed under a BZ-X710 microscope (Keyence, Osaka, Japan).

### Immunohistochemical analysis

IHC scoring was based on the percentage of positive cells. The staining intensity was scored as 0 (negative), 1+ (weak), 2+ (medium) or 3+ (strong). The percentage of stained cells was categorized as: 1, 0–10%; 2, 11–50%; and 3, more than 50% stained cells. The total IHC score was determined by multiplying the frequency and intensity scores^10^.

### Ethical approval

All procedures in the animal studies were performed in accordance with the institutional ethical standards in compliance with the ARRIVE guidelines (http://www.nc3rs.org.uk/page.asp?id=1357) and all relevant guidelines and regulations. This article does not contain any studies with human participants performed by any of the authors.

### Statistical analysis

Comparisons between two different groups were performed using Student’s *t* test. Comparisons between multiple groups were performed using one-way analysis of variance (ANOVA) followed by the Tukey–Kramer method. Statistical differences among means were considered significant when *p* < 0.05.

### Research involving human participants and/or animals

All aspects of the experimental design and procedure were reviewed and approved by the institutional ethics and animal welfare committees of Kobe University.

## Results

### Nanaomycin K inhibited the cell growth of bladder cancer cells under EMT induced by TGF-β

**Figure 1:**
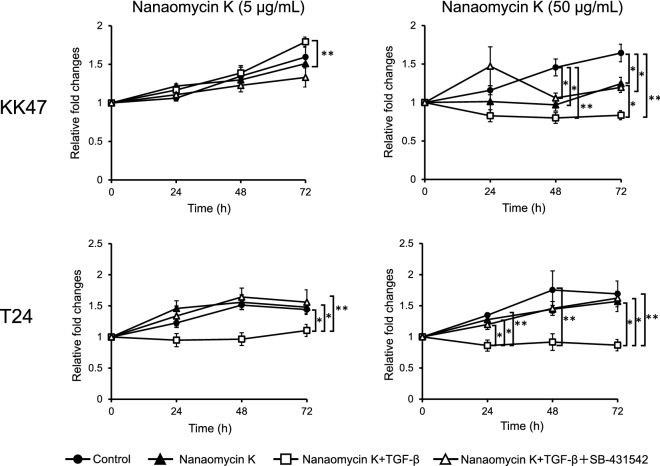
Cell cytotoxicity of nanaomycin K against KK47 and T24 in vitro. In vitro cell proliferation assays in KK47 and T24 cell lines were treated with 5 µg/mL nanaomycin K or 50 µg/mL nanaomycin K, with or without TGF-β, and with or without SB-431542 for 72 h. Control cells were treated with DMSO (vehicle control) (n = 3, average ± SE bars, *p < 0.05, **p < 0.01). Graphs show the relative fold changes of cell proliferation normalized by that at 0 h culture.

Both 5 µg/mL and 50 µg/mL of nanaomycin K significantly inhibited KK47 and T24 cancer cell growth in the presence of TGF-β compared to controls after 72 h of culture (*p* < 0.01, or *p* < 0.01) (Fig. 1). The higher concentration of nanaomycin K showed early cell cytotoxicity and strongly inhibited cell growth at 72 h in vitro. In contrast, nanaomycin K did not significantly inhibit cell growth in culture without TGF-β or with TGF-β with SB-431542, a TGF-β receptor inhibitor, suggesting that the observed cytotoxicity depended on TGF-β signaling.

### Migration inhibitory effect of nanaomycin K

**Figure 2:**
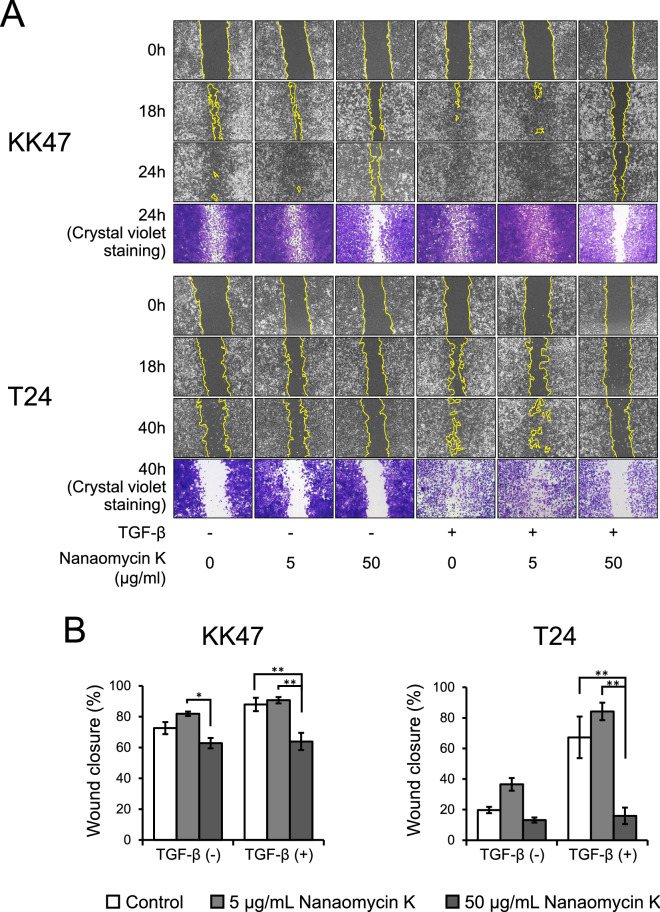
Wound healing assay. Invasion and migration ability was investigated in the presence of nanaomycin K (5 µg/mL or 50 µg/mL) with or without TGF-β in KK47 and T24 cell lines (100 ×) up to 40 h cultures (**A**). Control cells were treated with DMSO (vehicle control). Representative figures are shown. Wound closures compared to the wound at 0 h were shown (**B**) (n = 4, average ± SE bars, *p < 0.05, **p < 0.01).

TGF-β promoted wound closure in both KK47 and T24 cells. A low concentration (5 µg/mL) of nanaomycin K did not inhibit wound closure in either cell line with or without TGF-β, but a high concentration (50 µg/mL) of nanaomycin K significantly inhibited wound closure in both KK47 and T24 in the presence of TGF-β, 24 h or 40 h culture after scratching (*p* < 0.05, *p* < 0.01) (Fig. 2A,B). These results suggested that nanaomycin K inhibited the cell migration driven by TGF-β.

### Changes in EMT properties after culture with nanaomycin K

**Figure 3:**
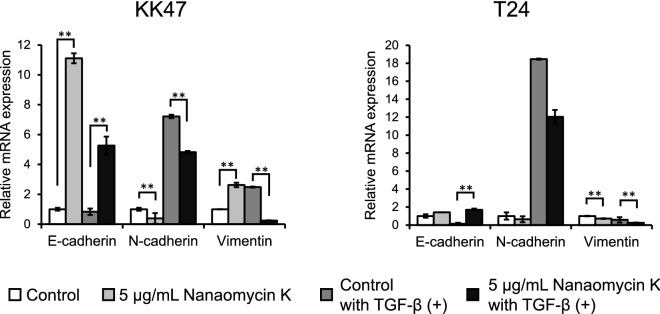
Gene expression of EMT markers induced by nanaomycin K in vitro. KK47 and T24 cells were treated with 5 µg/mL nanaomycin K with or without TGF-β in vitro for 48 h and the gene expressions of E-cadherin, N-cadherin and vimentin were determined by real time RT-PCR. Control cells were treated with DMSO (vehicle control) and mRNA levels were standardized by the expression levels of control gene β-actin (n = 3, average ± SE bars, *p < 0.05, **p < 0.01).

In the presence of TGF-β, nanaomycin K significantly decreased the gene expression of N-cadherin (*p* < 0.01) and vimentin (*p* < 0.01), and increased E-cadherin expression (*p* < 0.01) (Fig. 3). Without TGF-β, nanaomycin K significantly increased the expression of E-cadherin and decreased N-cadherin expression (*p* < 0.01) in KK47 cells. In T24 cells, nanaomycin K significantly decreased vimentin (*p* < 0.01) and increased E-cadherin expression (*p* < 0.01) in the presence of TGF-β. Nanaomycin K also inhibited EMT without TGF-β, but significance was seen only in vimentin (*p* < 0.01), indicating that T24 already had mesenchymal phenotypes. These results suggested that nanaomycin K inhibited EMT and induced epithelial properties in bladder cancer cell lines.

### Expression of EMT-related protein and MAPK signaling after culture with nanaomycin K

**Figure 4:**
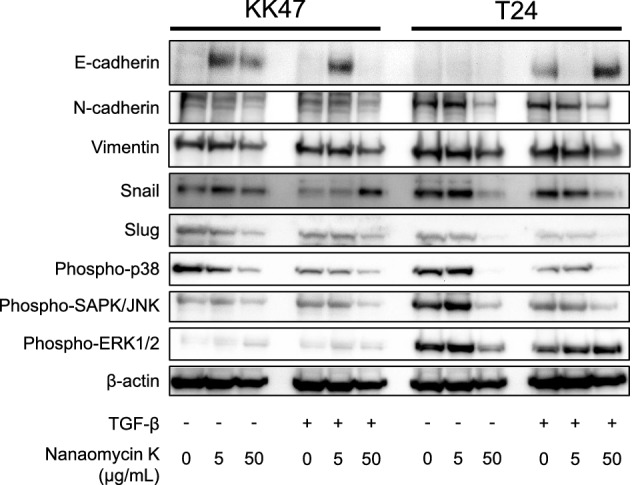
Western blotting for EMT and MAPK signaling. The expressions of E-cadherin, N-cadherin, vimentin, Snail, Slug, phopho-p38, phospho-SAPK/JNK, phospho-ERK1/2, and β-actin were determined in the presence of nanaomycin K (5 µg/mL or 50 µg/mL) with or without TGF-β in vitro for 48 h in KK47 and T24 cells. Full-length blots are presented in Supplementary Figure S1.

A low concentration (5 µg/mL) of nanaomycin K increased the expression of E-cadherin in KK47 without TGF-β while a high concentration (50 µg/mL) of nanaomycin K increased E-cadherin expression in T24 in the presence of TGF-β (Fig. 4). N-cadherin and vimentin were decreased after treatment with a high concentration of nanaomycin K both in cell lines.

The expression of Snail, a family of transcription factors that induce EMT, was apparently decreased in T24 by high concentrations of nanaomycin K independently of TGF-β stimulation, but a similar effect was not seen in KK47. High concentrations of nanaomycin K remarkably decreased the expression of Slug in KK47 and T24 independently of TGF-β stimulation.

Regarding the MAPK signaling pathway, a high concentration of nanaomycin K remarkably decreased the expression of phospho-p38 and phospho-SAPK/JNK in both KK47 and T24 cells, but not phospho-ERK1/2. In particular, more intensive EMT and MAPK inhibition by nanaomycin K was seen in T24 cells compared to KK47.

### Detection of apoptosis induced by nanaomycin K

**Figure 5:**
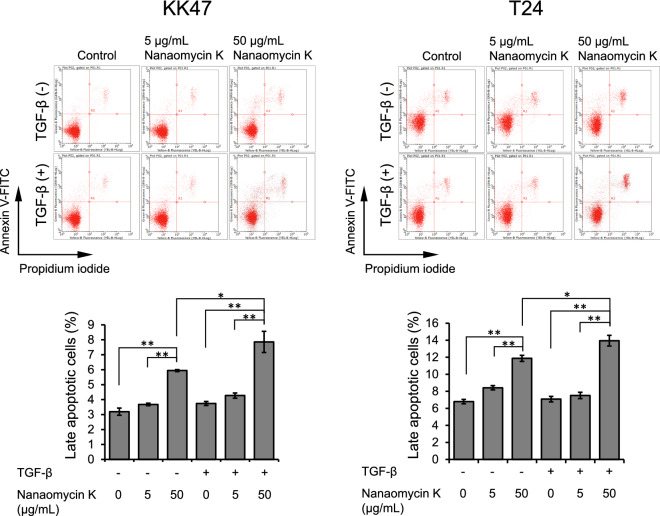
Apoptosis detection. KK47 and T24 cells were treated with nanaomycin K (5 µg/mL or 50 µg/mL) with or without TGF-β in vitro for 48 h and the proportions of late apoptotic cells were detected by Annexin-V-FITC and propidium iodide (PI) (n = 3, average ± SE bars, *p < 0.05, **p < 0.01). Representative dot plots of each treatment are shown. Data were analyzed by InCyte software (version 3.1, https://www.luminexcorp.com/ja/guava-easycyte-flow-cytometers/#software) (Luminex).

High concentration (50 µg/mL) of nanaomycin K induced significantly higher populations of late apoptotic cells compared to controls and low concentrations (5 µg/mL) of nanaomycin K in both KK47 and T24 cells after 48 h treatment (*p* < 0.01). Apoptosis induction was significantly augmented by TGF-β (p < 0.05) (Fig. 5). These results suggested that nanaomycin K strongly induced apoptosis in cells undergoing EMT.

### Nanaomycin K inhibited tumor growth in vivo

**Figure 6:**
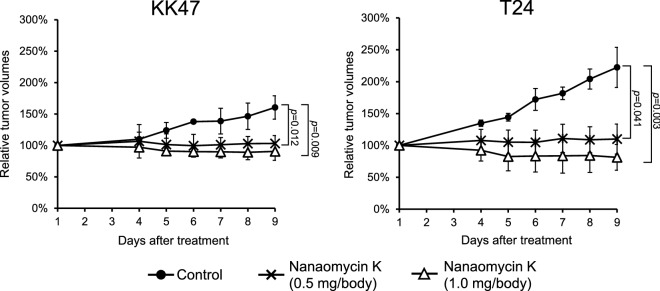
In vivo tumor inhibitory effects of nanaomycin K. Two bladder cancer cell lines, KK47 and T24, were subcutaneously inoculated into BALB/c nu/nu mice. After tumor growth was confirmed, mice were intratumorally treated with 0.5 mg/body nanaomycin K, 1.0 mg/body nanaomycin K, or vehicle control at day 1 (n = 4). After treatment, the tumor volume was measured for 9 days. The tumor volume of each mouse was standardized by that at day 1 and the tumor growth ratio is shown in the graphs (n = 4, average ± SE bars).

Intratumoral administration of nanaomycin K at both 0.5 mg and 1.0 mg/body significantly inhibited KK47 tumor growth after 9 days of treatment compared to controls (*p* = 0.012 and *p* = 0.009, respectively) (Fig. 6). No obvious adverse events were observed in either group after treatment. In T24 bladder cancer, both low and high doses of nanaomycin K significantly inhibited tumor growth 9 days after treatment compared to controls (*p* = 0.041 and *p* = 0.003, respectively), but in both tumor models the higher concentration of nanaomycin K completely suppressed tumor growth, suggesting that anti-tumor effects of nanaomycin K are dose-dependent.

### Changes of EMT properties and cell proliferation in tumor tissues after treatment with nanaomycin K

**Figure 7:**
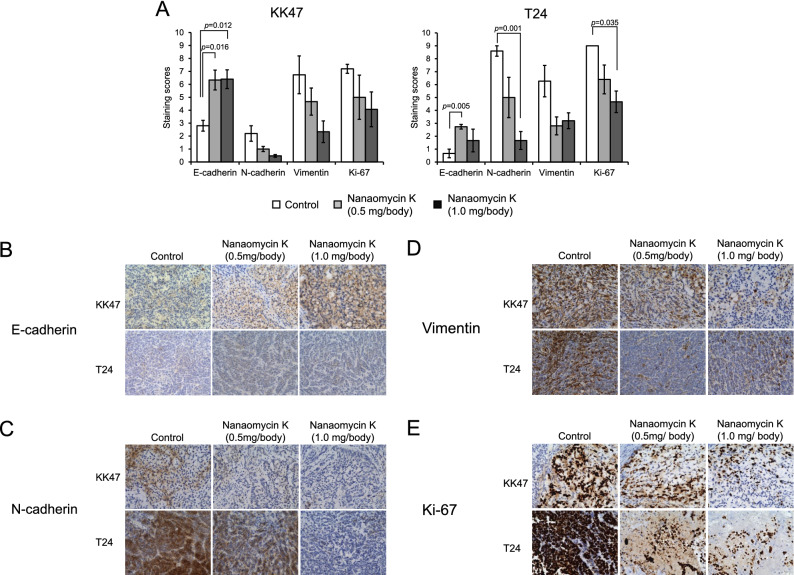
(**A**–**E**) Immunohistochemical analysis of KK47 and T24 mouse tumors for EMT markers and Ki-67 after nanaomycin K treatment. Marker expressions of E-cadherin, N-cadherin, vimentin and Ki-67 in tumor tissues were determined by immunohistochemical staining and evaluated by staining score (0–9) (**A**). Representative images for each marker is shown in (**B**) E-cadherin, (**C**) N-cadherin, (**D**) vimentin, (**E**) Ki-67, respectively.

The expression of E-cadherin was significantly increased by both 0.5 mg/body and 1 mg/body doses of nanaomycin K compared with control mice in KK47 (*p* = 0.016 and *p* = 0.012, respectively). In T24 tumor, 0.5 mg/body nanaomycin K significantly increased the expression of E-cadherin (*p* = 0.005). The results of IHC scoring were shown Fig. 7A. Representative pictures are shown in Fig. 7B–E. N-cadherin expression were decreased in both tumors, but statistical significance was seen only in T24 treated by 1 mg/body of nanaomycin K (*p* = 0.001). The expression of vimentin was also decreased by nanaomycin K in both tumors, but no statistical significance was seen. Expression of Ki-67, a cell proliferation marker, was significantly decreased by nanaomycin K in T24 (*p* = 0.035) (Fig. 7A,E). These results suggested that intratumoral administration of nanaomycin K inhibited EMT and tumor growth, especially in muscle-invasive bladder cancer.

## Discussion

In this study, we investigated the anti-tumor activity and EMT inhibitory effect of nanaomycin K against two bladder cancer cell lines, one muscle-invasive and one non-muscle invasive, in vitro and in vivo*.* Nanaomycin K, isolated from a cultured broth of *S. rosa* subsp. *notoensis* OS-3966^T^, is a new nanaomycin analog with an ergothioneine moiety in the partial structure^4^. It has stronger bioactivity for killing MDCK cells undergoing EMT than nanaomycin H. Therefore, in this study we explored the bioactivity of nanaomycin K as an EMT inhibitor in bladder cancer cells, where EMT plays a major role in invasion, migration and cancer progression.

In our in vitro study showed that nanaomycin K significantly inhibited tumor proliferation, invasion and migration via EMT in non-muscle invasive KK47 and muscle-invasive T24 cancer cells, especially in the presence of TGF-β. Nanaomycin K showed dose-dependent cell cytotoxicity when cells were stimulated by TGF-β. Many studies have demonstrated that TGF-β family members are potent initiators of EMT in cancer cells^13,14^. We also demonstrated that SB-431542, a TGF-β signaling inhibitor, suppressed the inhibitory effect of nanaomycin K on cancer cell proliferation in vitro. SB-431542 is a known potent selective inhibitor of TGF-β receptors ALK5 and ALK7, which are responsible for the phosphorylation of Smad2^15^. These findings indicated that nanaomycin K was specifically cytotoxic to cancer cells undergoing EMT in response to TGF-β stimulation.

Cancer cell invasion and migration have important roles in cancer metastasis associated with EMT. Wound healing assay showed that nanaomycin K significantly inhibited the wound healing of KK47 and T24 cells stimulated with TGF-β, suggesting that nanaomycin K had an inhibitory effect on cell migration. TGF-β induced EMT and invasiveness in bladder cancer cells as an EMT model by proteomics analysis^16^. Aggressive tumors such as T24 often accelerate cell motility, invasion and survival by inducing EMT through TGF-β in an autocrine and paracrine manner^17^, supporting the finding that T24 had already acquired mesenchymal cell phenotypes.

Regarding EMT-related genes and proteins, we demonstrated that nanaomycin K significantly increased the expression of E-cadherin in both KK47 and T24, especially when the cells were treated with TGF-β. At the protein level, nanaomycin K remarkably increased the expression of E-cadherin and decreased N-cadherin and vimentin expression in both cell lines. We also demonstrated that Snail and Slug, both transcription factors involved in EMT and wound healing^18^ were apparently decreased by nanaomycin K, especially in T24, supporting the conclusion that nanaomycin K reversed EMT and altered the cancer cells to acquire an epithelial cell phenotype.

At the molecular level, EMT is characterized by loss of E-cadherin. Once wound healing occurs, EMT facilitates repair by promoting epithelial migration to the site of injury. Then, when repair starts, epithelial cells re-express E-cadherin via a process of mesenchymal-to-epithelial transition (MET)^8^. EMT promoted stemness involving the overexpression of SOX2 and NANOG in muscle-invasive bladder cancer clinical samples. Islam et al. also reported that the EMT induced by TGF-β possibly correlated with the increased stemness phenotype in bladder cancer, and suggested that EMT inhibition may be a possible target to reverse the stemness^19^. These reports support the conclusion that nanaomycin K reverses TGF-β-induced EMT and possibly stemness in bladder cancer cells.

We experimentally observed later cell apoptosis was induced by nanaomycin K in both KK47 and T24 cells, especially when cells were stimulated with TGF-β. The roles of TGF-β in cell apoptosis, vary during cancer progression and remain very controversial in bladder cancer tumorigenesis. At later apoptosis stages, when cancer cells have undergone oncogenic mutation and/or have lost tumor suppressor gene functions, TGF-β has a role as a promotor by stimulating tumor cells to undergo EMT^6^. Our data demonstrated that nanaomycin K specifically killed bladder cancer cells which were undergo EMT by induction of late apoptosis.

We also found that nanomycin K decreased the protein expression of phospho-p38 and phospho-SAPK/JNK but not phospho-ERK1/2 in T24 cells. Reportedly, ERK1/2, JNKs, and p38, in the MAPK signaling pathway family, regulate EMT in bladder tissues, and the increase of those proteins significantly correlated with EMT in bladder cells^20^. Our findings suggested that nanaomycin K could inhibit EMT thorough the suppression of JNK and the p38 pathway in muscle-invasive bladder cancer cells. More mechanistic studies are needed to reveal which signaling pathways canonically affect the cancer cell cytotoxicity and cell apoptosis by nanaomycin K.

Our in vivo studies showed that intratumoral administration of nanaomycin K significantly inhibited both KK47 and T24 tumor growth in a dose-dependent fashion in mice xenografts. Consistent with the in vitro studies, nanaomycin K elicited much stronger tumor growth inhibition effects against T24, suggesting that nanaomycin K is more effective in muscle-invasive bladder cancer. In immunohistochemical staining, the expression of E-cadherin was significantly increased while expression of both N-cadherin and vimentin was remarkably decreased by nanaomycin K treatment in both KK47 and T24 tumors. Ki-67, a proliferation marker for cancer cells, was especially significantly decreased in T24 tumors by nanaomycin K treatment. High Ki-67 expression in clinical bladder cancer tissues is associated with cancer progression and poor outcomes in both non-muscle-invasive and muscle-invasive cancers^21,22^. Our results suggested that nanaomycin K directly reversed EMT and inhibited the cell proliferation of muscle invasive bladder cancer cells and exhibited cytotoxicity to cancer cells undergoing EMT.

Similar to our study, Liang et al. found that ablation of TGF-β signaling by a TGF-β receptor 1 inhibitor, LY364947, inhibited cancer cell proliferation, cancer stem cell population and EMT, and suppressed cancer progression in an orthotopic bladder cancer mouse model^23^. They also found that TGF-β signaling is important for the invasive and metastatic process in bladder cancer, including increased expression of Ki-67 and anti-apoptosis effects. Their findings support our results that nanaomycin K inhibits TGF-β signaling and suppresses EMT in bladder cancer cells in vivo.

There are no conclusive anti-EMT drugs for advanced bladder cancer. Immune checkpoint inhibitors such as pembrolizumab have been approved for advanced bladder cancer treatment, and therefore combinational therapy using immune checkpoint inhibitors with other drugs may turn out to be effective treatments^24^. Nanaomycin K may also be a possible anti-EMT drug candidate in combination with immune checkpoint inhibitors.

We would like to emphasize the study limitations. First, we only used two bladder cancer cell lines to investigate the anti-tumor effect of nanaomycin K. Second, the mechanism of apoptosis induced by nanaomycin K should be investigated to reveal which pathways are responsible for the apoptosis. Third, discrepancy between the results of gene expression and protein expression related EMT should be investigated in the future study.

In conclusion, we demonstrated that nanaomycin K had significant anti-EMT and anti-tumor effects in bladder cancer cells. Our findings suggest that nanaomycin K may be a therapeutic candidate for advanced bladder cancer. Further in vivo and in vitro studies are needed to investigate the mechanisms of action of nanaomycin K.

## Supplementary Information

Supplementary Figure S1.

## References

[1] Omura S, Tanaka H, Koyama Y, Oiwa R, Katagiri M (1974). Nanaomycins A and B, new antibiotics produced by a strain of *Streptomyces*. J. Antibiot. (Tokyo).

[2] Nakashima T (2015). New compounds, nanaomycin F and G, discovered by physicochemical screening from a culture broth of *Streptomyces**rosa* subsp. *notoensis* OS-3966. J. Biosci. Bioeng..

[3] Nakanishi J (2019). An application of photoactivatable substrate for the evaluation of epithelial–mesenchymal transition inhibitors. Anal. Sci..

[4] Matsuo H (2019). Nanaomycin K, a new epithelial–mesenchymal transition inhibitor produced by the actinomycete “*Streptomyces**rosa* subsp. *notoensis*” OS-3966. J. Biosci. Bioeng..

[5] Pearson GW (2019). Control of invasion by epithelial-to-mesenchymal transition programs during metastasis. J. Clin. Med..

[6] Hao Y, Baker D, Ten Dijke P (2019). TGF-β-mediated epithelial–mesenchymal transition and cancer metastasis. Int. J. Mol. Sci..

[7] Knollman H (2015). Muscle-invasive urothelial bladder cancer: An update on systemic therapy. Ther. Adv. Urol..

[8] McConkey DJ (2009). Role of epithelial-to-mesenchymal transition (EMT) in drug sensitivity and metastasis in bladder cancer. Cancer Metastasis Rev..

[9] van Staalduinen J, Baker D, Dijke PT, van Dam H (2018). Epithelial–mesenchymal-transition-inducing transcription factors: New targets for tackling chemoresistance in cancer?. Oncogene.

[10] Kitagawa K (2019). Possible correlation of sonic hedgehog signaling with epithelial–mesenchymal transition in muscle-invasive bladder cancer progression. J. Cancer Res. Clin. Oncol..

[11] Kawata M (2012). TGF-β-induced epithelial–mesenchymal transition of A549 lung adenocarcinoma cells is enhanced by pro-inflammatory cytokines derived from RAW 264.7 macrophage cells. J. Biochem..

[12] Liao S (2019). Long non-coding RNA H19 promotes the proliferation and invasion of lung cancer cells and regulates the expression of E-cadherin, N-cadherin, and vimentin. Onco Targets Ther..

[13] Syed IS, Pedram A, Farhat WA (2016). Role of sonic Hedgehog (Shh) signaling in bladder cancer stemness and tumorigenesis. Curr. Urol. Rep..

[14] Stojnev S (2019). Prognostic impact of canonical TGF-β signaling in urothelial bladder cancer. Medicina (Kaunas).

[15] DaCosta BS, Major C, Laping NJ, Roberts AB (2004). SB-505124 is a selective inhibitor of transforming growth factor-beta type I receptors ALK4, ALK5, and ALK7. Mol. Pharmacol..

[16] Yang G (2016). Quantitative analysis of differential proteome expression in epithelial-to-mesenchymal transition of bladder epithelial cells using SILAC method. Molecules.

[17] Khin SS (2009). BAMBI gene is epigenetically silenced in subset of high-grade bladder cancer. Int. J. Cancer.

[18] Ganesan R, Mallets E, Gomez-Cambronero J (2016). The transcription factors Slug (SNAI2) and Snail (SNAI1) regulate phospholipase D (PLD) promoter in opposite ways towards cancer cell invasion. Mol. Oncol..

[19] Islam SS (2016). Sonic hedgehog (Shh) signaling promotes tumorigenicity and stemness via activation of epithelial-to-mesenchymal transition (EMT) in bladder cancer. Mol. Carcinog..

[20] Yu D (2017). Cigarette smoke induced urocystic epithelial mesenchymal transition via MAPK pathways. Oncotarget.

[21] Thakur B, Kishore S, Dutta K, Kaushik S, Bhardwaj A (2017). Role of p53 and Ki-67 immunomarkers in carcinoma of urinary bladder. Indian J. Pathol. Microbiol..

[22] Ko K, Jeong CW, Kwak C, Kim HH, Ku JH (2017). Significance of Ki-67 in non-muscle invasive bladder cancer patients: A systematic review and meta-analysis. Oncotarget.

[23] Liang Y (2016). Conditional ablation of TGF-β signaling inhibits tumor progression and invasion in an induced mouse bladder cancer model. Sci. Rep..

[24] Lopez-Beltran A (2021). Immune checkpoint inhibitors for the treatment of bladder cancer. Cancers (Basel).

