# Supplementary material for: Nanaomycin K inhibited epithelial mesenchymal transition and tumor growth in bladder cancer cells in vitro and in vivo
Source: Sci Rep. 2021 Apr 28;11:9217. doi: 10.1038/s41598-021-88741-3 (PMC8080577; doi:10.1038/s41598-021-88741-3)
Supplement: Supplementary file 1 — Supplementary Figure S1. [file 41598_2021_88741_MOESM1_ESM.docx]

**Nanaomycin K inhibited epithelial mesenchymal transition and tumor growth in bladder cancer cells *in vitro* and *in vivo***

Koichi Kitagawa^1,2*^, Katsumi Shigemura^2,3†^, Aya Ishii^2*^, Takuji Nakashima^4^, Hirotaka Matsuo^4^, Yoko Takahashi^4^, Satoshi Omura^4^, Jun Nakanishi^5^, and Masato Fujisawa^3^

*: These authors contributed equally.

^1^Department of Advanced Medical Science, Kobe University Graduate School of Science, Technology and Innovation, 7-5-1 Kusunoki-cho, Chuo-ku, Kobe, 650-0017, Japan.

^2^Department of Public Health, Kobe University Graduate School of Health Sciences, 7-10-2 Tomogaoka, Suma-ku, Kobe, 654-0142, Japan.

^3^Department of Urology, Kobe University Graduate School of Medicine, 7-5-2 Kusunoki-cho, Chuo-ku, Kobe, Hyogo 650-0017, Japan.

^4^Kitasato Institute for Life Sciences, Kitasato University, 5-9-1 Shirokane, Minato-ku, Tokyo 108-8641, Japan; Graduate School of Infection Control Sciences, Kitasato University, 5-9-1 Shirokane, Minato-ku, Tokyo 108-8641, Japan.

^5^Research Center for Functional Materials, National Institute for Materials Science (NIMS), 1-1 Namiki, Tsukuba, Ibaraki 305-0044, Japan.

**†Correspondence address**

Katsumi Shigemura, MD, PhD

Department of Urology, Kobe University Graduate School of Medicine,

7-5-1 Kusunoki-Cho, Chuo-Ku, Kobe, Japan 650-0017

Telephone; 81-78-382-6155, Fax; 81-78-382-6169

E-mail address: katsumi@med.kobe-u.ac.jp

Supplementary Fig. S1. Uncropped images and cropped area of western blot.
